# The Microtubule-Stabilizing Protein CLASP1 Associates with the *Theileria annulata* Schizont Surface via Its Kinetochore-Binding Domain

**DOI:** 10.1128/mSphere.00215-17

**Published:** 2017-08-23

**Authors:** Sandra Huber, Romina Theiler, Daniel de Quervain, Olga Wiens, Tulin Karangenc, Volker Heussler, Dirk Dobbelaere, Kerry Woods

**Affiliations:** a Institute for Animal Pathology, Vetsuisse Faculty, University of Bern, Bern, Switzerland; b Institute for Molecular Pathobiology, Vetsuisse Faculty, University of Bern, Bern, Switzerland; c Department of Parasitology, Adnan Menderes University, Faculty of Veterinary Medicine, Isikli, Aydin, Turkey; d Institute of Cell Biology, University of Bern, Bern, Switzerland; University at Buffalo

**Keywords:** *Theileria*, apicomplexan parasites, microtubule, BioID, CLASP1, cytoskeleton

## Abstract

*Theileria* is an apicomplexan parasite whose presence within the cytoplasm of a leukocyte induces cellular transformation and causes uncontrolled proliferation and clonal expansion of the infected cell. The intracellular schizont utilizes the host cell’s own mitotic machinery to ensure its distribution to both daughter cells by associating closely with microtubules (MTs) and incorporating itself within the central spindle. We show that CLASP1, an MT-stabilizing protein that plays important roles in regulating kinetochore-MT attachment and central spindle positioning, is sequestered at the *Theileria annulata* schizont surface. We used live-cell imaging and immunofluorescence in combination with MT depolymerization assays to demonstrate that CLASP1 binds to the schizont surface in an MT-independent manner throughout the cell cycle and that the recruitment of the related CLASP2 protein to the schizont is MT dependent. By transfecting *Theileria-*infected cells with a panel of truncation mutants, we found that the kinetochore-binding domain of CLASP1 is necessary and sufficient for parasite localization, revealing that CLASP1 interaction with the parasite occurs independently of EB1. We overexpressed the MT-binding domain of CLASP1 in parasitized cells. This exhibited a dominant negative effect on host MT stability and led to altered parasite size and morphology, emphasizing the importance of proper MT dynamics for *Theileria* partitioning during host cell division. Using coimmunoprecipitation, we demonstrate that CLASP1 interacts, directly or indirectly, with the schizont membrane protein p104, and we describe for the first time TA03615, a *Theileria* protein which localizes to the parasite surface, where it has the potential to participate in parasite-host interactions.

**IMPORTANCE** *T. annulata*, the only eukaryote known to be capable of transforming another eukaryote, is a widespread parasite of veterinary importance that puts 250 million cattle at risk worldwide and limits livestock development for some of the poorest people in the world. Crucial to the pathology of *Theileria* is its ability to interact with host microtubules and the mitotic spindle of the infected cell. This study builds on our previous work in investigating the host and parasite molecules involved in mediating this interaction. Because it is not possible to genetically manipulate *Theileria* schizonts, identifying protein interaction partners is critical to understanding the function of parasite proteins. By identifying two *Theileria* surface proteins that are involved in the interaction between CLASP1 and the parasite, we provide important insights into the molecular basis of *Theileria* persistence within a dividing cell.

## Abstract (precis)

*T. annulata*, the only eukaryote known to be capable of transforming another eukaryote, is a widespread parasite of veterinary importance that puts 250 million cattle at risk worldwide and limits livestock development for some of the poorest people in the world. Crucial to the pathology of *Theileria* is its ability to interact with host microtubules and the mitotic spindle of the infected cell. This study builds on our previous work in investigating the host and parasite molecules involved in mediating this interaction. Because it is not possible to genetically manipulate *Theileria* schizonts, identifying protein interaction partners is critical to understanding the function of parasite proteins. By identifying two *Theileria* surface proteins that are involved in the interaction between CLASP1 and the parasite, we provide important insights into the molecular basis of *Theileria* persistence within a dividing cell.

## INTRODUCTION

*Theileria annulata* is a tick-borne parasite of the apicomplexan phylum. This parasite causes tropical theileriosis, a severe disease in cattle that is prevalent in the Mediterranean, the Middle East, India, and the Far East. *T. annulata* infects predominantly bovine B cells and bovine macrophages (BoMac) (1, 2), and within 15 to 30 min of invasion, the parasite dissolves the surrounding host cell membrane and establishes its niche within the host cell cytoplasm (3). Host cell microtubules (MTs) are rapidly recruited to the invasive sporozoite and remain closely associated with the parasite as it differentiates into a multinucleated schizont (3, 4).

Many intracellular apicomplexan parasites manipulate the signaling pathways of their host cell to favor their own survival (5). *Theileria* modifies its host to an impressive extent and is the only eukaryotic cell known to induce the transformation of another cell. The presence of the schizont confers a cancer-like phenotype to the infected cell, inducing antiapoptotic signaling (6) and uncontrolled proliferation (7, 8). Infected cells become highly motile and invasive, facilitating the spread of the parasite throughout the animal and contributing to pathogenesis (9–12). The transformed phenotype conferred upon the infected host is reversible upon killing of the parasite, making *Theileria-*infected cells a unique model in which to study leukocyte cell signaling pathways related to proliferation and survival (8).

The *Theileria* schizont is strictly intracellular and ensures its own persistence within the continually dividing infected cell by coopting the host’s own mitotic apparatus. Host cell astral and central spindle MTs become stably associated with the schizont surface, and we previously demonstrated that disrupting this interaction prevents faithful parasite segregation during host cell cytokinesis (13). Host cell polo-like kinase 1 (Plk1), a molecule with key regulatory roles in mitosis and cytokinesis, was found to coat the schizont surface during host cell G_2_ phase and anaphase, and its kinase activity is required to recruit central spindles to the parasite surface (13). We then demonstrated that the MT plus-end-tracking protein (+TIP) end-binding protein 1 (EB1) binds to the parasite surface, where it likely plays a role in directing growing MTs to the schizont surface (14). +TIPs are a large and diverse group of MT-associated proteins (MAPs) that accumulate at the plus ends of growing MTs, with members of the family possessing MT-polymerizing, -depolymerizing, or -stabilizing functions (reviewed in reference 15). +TIPs regulate MT dynamics by interacting with MTs, and with one another, in a complex and dynamic interaction network, and they have functions in chromosome segregation, cell polarization, cell migration, organelle transport, and intracellular signaling. We showed that the *T. annulata* schizont membrane protein p104 (locus tag TA08425) possesses a typical EB1-binding SxIP motif and that it interacts specifically with host cell EB1. EB1 interacts directly with MT plus ends (16) and with almost all other +TIPs tested to date (17). EB1 is therefore often referred to as the master regulator of MT dynamics. The recruitment of EB1 to the parasite surface thus prompted us to test the localization of other EB1-binding +TIPs in *Theileria-*infected cells, particularly those with known MT-stabilizing properties. Our aim is to more fully understand how *Theileria* ensures its distribution to both daughter cells during host cell cytokinesis, because this is crucial for maintaining cellular transformation and is thus central to the pathogenesis of this important parasite.

The CLIP-170-associating protein 1 (CLASP1) is an MT-stabilizing +TIP that is involved in the regulation of MT-cortex interactions during interphase (18). CLASP1 is required for maintaining spindle position during cell division by ensuring the stabilization, or “capture,” of astral MTs at the cortex (19). CLASP1 and the closely related CLASP2 are known EB1-binding partners, with each possessing two SxIP motifs (17, 18). In mitotic cells, CLASP1 and CLASP2 associate with spindle poles, kinetochores, and MT plus ends within the spindle (20, 21), and CLASP1 was found to be a constituent of the outermost kinetochore region, to which it binds independently of MTs (20). CLASP1 also regulates kinetochore-MT dynamics at a temporal level by forming mutually exclusive complexes at the kinetochore with either the MT-stabilizing protein astrin or the MT-depolymerizing kinesin Kif2b (22).

In this work, we demonstrate for the first time that the host cell CLASP1 is sequestered by *T. annulata* and that this interaction is MT independent and mediated by its kinetochore-binding domain. Further, we show that the schizont membrane protein p104 not only interacts with EB1, as we showed before (14), but also is a direct or indirect interaction partner of CLASP1. We also provide an initial characterization of TA03615, a frequently associated in *Theileria* (FAINT) domain-containing schizont surface protein, and demonstrate that TA03615 is also coimmunoprecipitated with CLASP1 and p104.

## RESULTS

### CLASP1 localizes to the surfaces of *T. annulata* schizonts and sporozoites.

**FIG 1:**
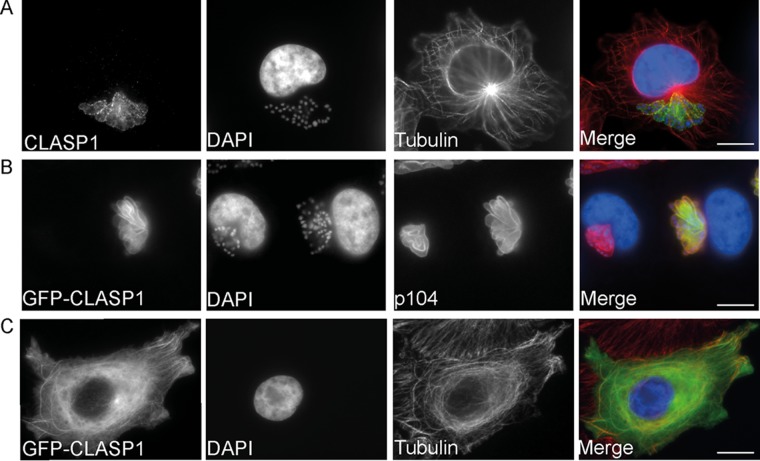
CLASP1 binds to the *Theileria* schizont surface. (A) Endogenous CLASP1 decorates the schizont surface in a punctate manner. *T. annulata-*transformed cells (TaC12) were indirectly labeled with rat anti-CLASP1 (green) and mouse antitubulin (red) antibodies and visualized by wide-field microscopy. Host and parasite nuclei are labeled with DAPI (blue). Scale bar, 10 μm. (B) GFP-CLASP1 is recruited to the parasite surface. TaC12 cells were transiently transfected with GFP-CLASP1 and analyzed 24 h posttransfection. The parasite surface is labeled with mouse anti-p104 antibodies (MAb 1C12) (red), and parasite and host cell nuclei are labeled with DAPI (blue). Scale bar, 10 μm. (C) GFP-CLASP1 localizes to microtubules in uninfected cells. Uninfected bovine macrophages (BoMac) were transfected with GFP-CLASP1 and analyzed 24 h posttransfection. Microtubules are labeled with mouse antitubulin antibodies (red), and DNA is labeled with DAPI. Scale bar, 10 μm.

Because host cell MTs stably associate with the schizont surface (13), we asked the question of whether the MT-stabilizing protein CLASP1 localizes to the parasite surface membrane in *T. annulata*-infected macrophages (TaC12). Immunofluorescence analysis (IFA) revealed that bovine CLASP1 indeed decorates the surface of a schizont in a punctate manner (Fig. 1A). The parasite was invariably coated with CLASP1 in every cell within the culture. CLASP1 is reported to localize to MT plus ends in HeLa cells in interphase (18) and to kinetochores, spindle poles, and the spindle midzone during mitosis (23). We detected all described localizations of CLASP1 in noninfected bovine macrophages (BoMac), confirming that the antibodies used in this study are specific (data not shown). We also confirmed the association of bovine CLASP1 with the parasite surface with a rabbit monoclonal anti-CLASP1 antibody (data not shown).

Overexpressing green fluorescent protein (GFP)-CLASP1 in TaC12 cells supported our observation that CLASP1 is recruited by the parasite (Fig. 1B). Upon overexpression in noninfected BoMac, GFP-CLASP1 localized along the lengths of MTs, as reported for other mammalian cell lines (24) (Fig. 1C), while in TaC12 cells, no MT localization was detected. GFP-CLASP1 is reported to track growing MT plus ends during interphase upon expression of low levels in HeLa cells (20), and we also observed the characteristic “comet-like” behavior of GFP-CLASP1 when it was expressed at low levels in BoMac (see Movie S1 in the supplemental material). Conversely, no such plus-end tracking was observed in the cytoplasm of transfected TaC12 cells (Movie S2), confirming that the sequestration of GFP-CLASP1 by the parasite is not an artifact caused by fixation.

10.1128/mSphere.00215-17.6 MOVIE S1  GFP-CLASP1 exhibits plus-end tracking in uninfected bovine macrophages (BoMac). Images were captured every 2 s for 2 min. Download MOVIE S1, MOV file, 17.2 MB. Copyright © 2017 Huber et al. 2017 Huber et al. This content is distributed under the terms of the Creative Commons Attribution 4.0 International license.

10.1128/mSphere.00215-17.7 MOVIE S2  GFP-CLASP1 is sequestered at the schizont surface when it is transiently expressed in TaC12 cells and exhibits no plus-end tracking in the host cytoplasm. Images were captured every second for 1 min. Download MOVIE S2, MOV file, 1.8 MB. Copyright © 2017 Huber et al. 2017 Huber et al. This content is distributed under the terms of the Creative Commons Attribution 4.0 International license.

Analysis of freshly infected peripheral blood mononuclear (PBM) cells revealed that CLASP1 associates with the sporozoite as early as 30 min postinfection (Fig. S1), suggesting that CLASP1 may be involved in the recruitment of MTs to the sporozoite surface during the establishment of infection. The TaC12 cell line has been in culture for many decades, and prolonged culture of *Theileria-*infected cells is associated with attenuated virulence and alterations in phenotype (25). To check whether CLASP1 association with the schizont is a general phenomenon associated with *Theileria* infection, we therefore also analyzed the localization of CLASP1 in a clonal line of *T. annulata*-infected PBM cells (clone TaD7) and in *Theileria parva*-infected T cells (Tp_951T_F31). We found CLASP1 to coat the schizont in both cell lines, suggesting that the sequestration of this host cell protein is likely to be a general feature of *Theileria* infection (Fig. S2).

10.1128/mSphere.00215-17.1 FIG S1  Host cell CLASP1 is recruited to the sporozoite rapidly following invasion of peripheral blood mononuclear (PBM) cells. PBM cells were infected with *T. annulata* A21/AT1 sporozoites and were fixed and analyzed with anti-CLASP1 (red) and anti-p104 (MAb 1C12) (green) antibodies 30 min postinfection (top). An uninfected cell is shown (bottom) for comparison. Host cells and sporozoite DNA were labeled with DAPI (blue). Scale bar, 10 μm. Download FIG S1, TIF file, 0.7 MB. Copyright © 2017 Huber et al. 2017 Huber et al. This content is distributed under the terms of the Creative Commons Attribution 4.0 International license.

10.1128/mSphere.00215-17.2 FIG S2  CLASP1 decorates the surfaces of *T. annulata* and *T. parva* schizonts in cloned cell lines. The clonal *T. annulata-*infected cell line TaD7 (top) and the *T. parva-*infected T cell line (Tp_951T_F31) (bottom) were prepared by cytospinning and analyzed with anti-CLASP1 antibodies (green for top panel, red in the bottom panel). The *T. annulata* schizont surface is labeled with anti-p104 (MAb 1C12) (red), and host and parasite nuclei are labeled with DAPI (blue). Download FIG S2, TIF file, 1.0 MB. Copyright © 2017 Huber et al. 2017 Huber et al. This content is distributed under the terms of the Creative Commons Attribution 4.0 International license.

### CLASP1 association with the *T. annulata* schizont surface is mediated by its kinetochore-binding domain.

**FIG 2:**
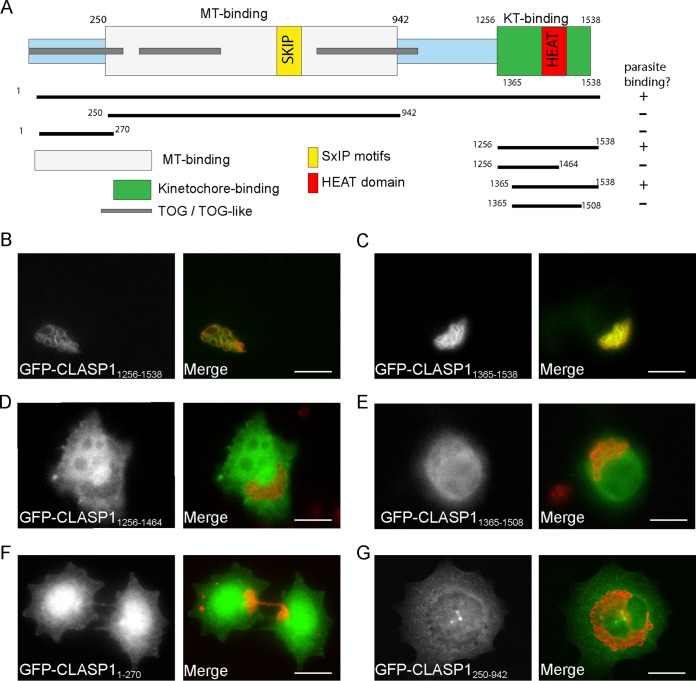
The kinetochore binding domain of CLASP1 is necessary and sufficient for parasite localization. (A) Schematic showing the different domains of human CLASP1. The MT-binding domain (aa 250 to 942) comprises two EB1-binding SxIP motifs (yellow), two TOG domains (aa 1 to 275, 284 to 552, gray), and a TOG-like domain (aa 833 to 1092, gray). The kinetochore (KT)-binding domain of CLASP1 (aa 1256 to 1538) is sufficient for parasite binding. The minimal parasite-binding domain (aa 1365 to 1538) of CLASP1 was determined by transient transfection of TaC12 cells with deletion constructs. (B to G) TaC12 cells were transiently transfected with plasmids encoding GFP-CLASP1_1256−1538_ (the kinetochore-binding domain) (B), GFP-CLASP1_1365–1538_ (C), GFP-CLASP1_1256–1464_ (D), GFP-CLASP1_1365–1508_ (E), GFP-CLASP1_1-250_ (the midbody-binding domain) (F), and GFP-CLASP1_250–942_ (the MT-binding domain) (G). In each case, the GFP signal is shown in gray. The merge shows the GFP signal (green) and the parasite labeled with rabbit anti-TaSP (red). In panels B to F, the scale bar represents 10 μm. In panel G the scale bar represents 25 μm.

In order to determine which domain of CLASP1 is responsible for targeting to the schizont, we tested a panel of CLASP1 truncation mutants, including the MT-binding domain (amino acids [aa] 250 to 942), the midbody-binding domain (aa 1 to 270), and the kinetochore-binding domain (aa 1256 to 1538) (20). We found that the kinetochore-binding domain (CLASP1_1256−1538_) was sufficient to localize to the parasite surface (Fig. 2A and B). This fragment was not reported to induce any dominant negative effects in HeLa cells and did not displace endogenous CLASP1 from kinetochores (20). In line with this, expression of GFP-CLASP1_1256−1538_ did not displace endogenous CLASP1 from the schizont surface (not shown) and could be used to track the parasite as infected host cells progressed through mitosis and cytokinesis (Movie S3). Further transfection experiments with a panel of truncation mutants revealed that 173 aa, corresponding to CLASP1_1365–1538_, is necessary and sufficient for parasite binding (Fig. 2C). Fragments comprising amino acids 1256 to 1464 (208 aa) (Fig. 2D) and 1365 to 1508 (143 aa) (Fig. 2E) failed to localize to the parasite surface, suggesting that the C terminus of CLASP1 is required to mediate the interaction. We could detect no colocalization of the midbody-binding domain (CLASP1_1−270_) with the parasite surface (Fig. 2F). We have previously shown that EB1 is recruited to the *Theileria* surface via a direct interaction with the SxIP motif embedded in the schizont membrane protein p104. Considering that the interaction between CLASP1 and EB1 is mediated via its two SxIP motifs (SKIP [aa 718 to 721] and SRIP [aa 741 to 744]), which are embedded within the MT-binding domain (CLASP1_250−942_), we considered it likely that the MT-binding domain is sufficient for parasite localization. We were therefore intrigued to see that, while CLASP1_250–942_ did indeed bind to MTs as reported (20), it did not colocalize with the schizont surface (Fig. 2G). These data show that CLASP1 sequestration by the schizont is not mediated via an interaction with EB1 but instead that the kinetochore-binding domain is necessary and sufficient for parasite localization.

10.1128/mSphere.00215-17.8 MOVIE S3  GFP-CLASP1_1256−1538_ causes no negative effect in cell cycle progression and can be used to label the surfaces of schizonts throughout the host cell cycle. Images were captured every 2 min for 3 h. Download MOVIE S3, MOV file, 12.5 MB. Copyright © 2017 Huber et al. 2017 Huber et al. This content is distributed under the terms of the Creative Commons Attribution 4.0 International license.

### Proper MT dynamics are required for maintaining the morphology and position of the intracellular schizont.

**FIG 3:**
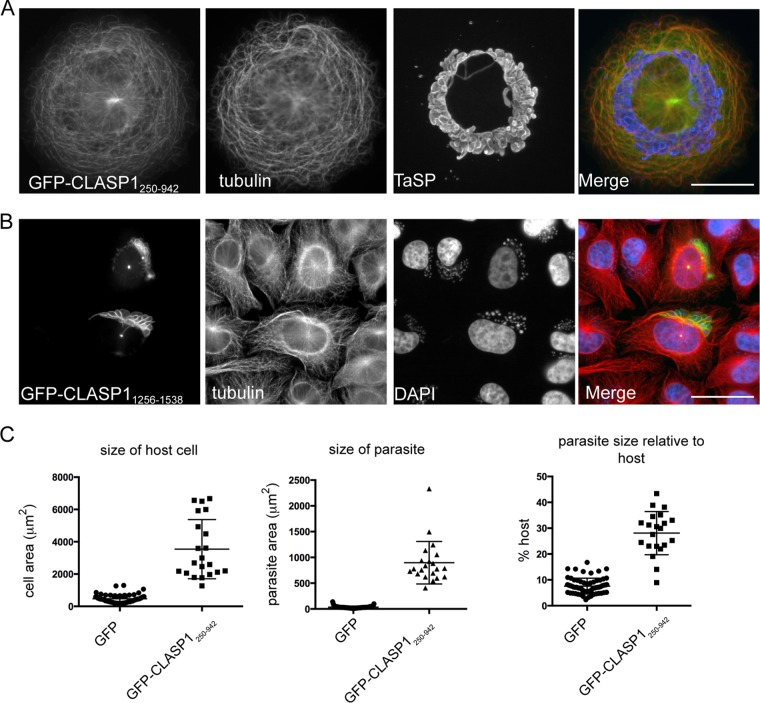
Proper MT dynamics are required for maintaining the morphology and position of the intracellular schizont. (A) TaC12 cells were transduced with the MT-binding domain of CLASP1, GFP-CLASP1_250–942_, and analyzed by wide-field microscopy. Cells were indirectly labeled with mouse antitubulin (red), and the parasite was surface labeled with rabbit anti-TaSP (blue). Scale bar, 25 μm. (B) TaC12 cells were transduced with GFP-CLASP1_1256−1538_ and analyzed by wide-field microscopy. Cells were indirectly labeled with mouse antitubulin (red), and host and parasite nuclei were labeled with DAPI (blue). Scale bar, 25 μm. (C) To assess the effect of expressing GFP-CLASP1_250–942_, the sizes (areas in square micrometers) of the host cell and parasite (first two graphs) were measured using Fiji and are represented as the parasite size relative to the host’s size (third graph). As a control, cells were transduced with GFP alone. *n =* 100.

The MT-binding domain of CLASP1 was reported to induce dominant negative effects in HeLa cells, where it induced the formation of highly stable MT arrays in interphase and caused severe mitotic defects when expressed during mitosis (20). Following transient transfection with GFP-CLASP1_250–942_, we noticed that transfected cells were frequently unusually large compared to nontransfected cells (note that the scale bar in Fig. 2G depicts 25 μm but that the scale bar in Fig. 2B to F represents 10 μm). To circumvent the problem of low transfection efficiency in *Theileria*-infected cells and to allow a quantitative analysis of the effect of GFP-CLASP1_250–942_ expression on *Theileria* morphology and positioning, we transduced TaC12 cells with lentiviral vectors expressing GFP-CLASP1_250–942_ (Fig. 3A). For comparison, TaC12 cells expressing GFP-CLASP1_1256−1538_ are shown (Fig. 3B). Again we observed that GFP-CLASP1_250–942_ localized to MT bundles, and both the host cell and parasite were large in comparison to cells expressing an empty vector control (Fig. 3A, quantified in panel C). The parasite often adopted a “circular” position within the cell, which is in striking contrast to the more usual position adopted by the schizont; it is generally oriented toward the host cell centrosome (compare Fig. 3A with Fig. 1A and B). This emphasizes the importance of the MT cytoskeleton in maintaining parasite positioning and is reminiscent of the observations made by Hulliger et al. (4), in whose study cells were treated with colchicine to disrupt microtubules. While host cell division stopped, parasite division continued, showing that the association of the parasite with MTs is not essential for parasite survival but rather for its segregation to daughter cells following cell division. We could not detect any mitotic cells expressing GFP-CLASP1_250–942_, even after following well-established protocols to arrest cells in mitosis (data not shown).

To further investigate the importance of a functional MT network for parasite morphology and positioning, we attempted to knock out CLASP1 from TaC12 cells using clustered regularly interspaced short palindromic repeat (CRISPR)/Cas9 technology. This attempt was unsuccessful, and the few clones that we obtained following selection still expressed CLASP1. A negative-selection genome-wide screen carried out using CRISPR/Cas9 analysis of haploid KBM7 cells indicated that CLASP1 may be an essential gene (26). We therefore did not pursue this approach further and instead made use of short hairpin RNA (shRNA) to deplete CLASP1 from parasitized cells. Using one set of shRNA primers, we could significantly (although not completely) deplete cellular CLASP1 levels (Fig. S3A and B). Following shRNA expression, we noted that cells were rather large and that the MT network was altered (notice the multiple microtubule-organizing centers [MTOCs] in knockdown cells) (Fig. S3A). Considering that our interest is to analyze the importance of CLASP1 function on *Theileria* behavior in infected macrophages, we searched for CLASP1-depleted cells undergoing cytokinesis. In such cells, the parasite was invariably normally distributed, and segregation to daughter cells following cytokinesis was not disrupted (Fig. S3C shows a mixed population of cells, with one cell expressing normal levels of CLASP1 next to a CLASP1-depleted cell undergoing cytokinesis). From these observations, we conclude that normal levels of CLASP1 are not essential for parasite association with host cell MTs and proper partitioning of the parasite during host cell cytokinesis. We suggest that it is likely that other host-parasite interactions on the parasite surface, including, for example, p104-EB1 (14) or TaSP-tubulin (27), compensate for a partial loss of CLASP1. Alternatively, we cannot exclude the possibility that small quantities of protein remaining following depletion are sufficient to retain CLASP1 protein function.

10.1128/mSphere.00215-17.3 FIG S3  Depletion of CLASP1 does not impact parasite segregation following host cell cytokinesis. (A) TaC12 cells were transduced three times with lentiviral particles delivering an shRNA sequence targeting bovine CLASP1 and then fixed for indirect immunofluorescence analysis. The top panel shows a wild-type culture; the bottom panel is a mixed CLASP1-shRNA population. Cells were labeled with anti-CLASP1 (green), antitubulin (DM1A) (red), and DAPI. Scale bar, 10 μm. (B) The wild-type and two CLASP1-shRNA populations were lysed and analyzed by Western blotting with anti-CLASP1 antibodies (top). Tubulin was used as a loading control. (C) A CLASP1-negative dividing cell is depicted alongside a CLASP1-positive cell and labeled with anti-CLASP1 (green), antitubulin (DM1A) (red), anti-TaSP (Cy5), and DAPI (blue). Merges of DAPI and CLASP1 and of DAP1, CLASP1, and tubulin are shown. Scale bar, 10 μm. Download FIG S3, TIF file, 2.9 MB. Copyright © 2017 Huber et al. 2017 Huber et al. This content is distributed under the terms of the Creative Commons Attribution 4.0 International license.

### CLASP1 binds to the schizont surface in an MT-independent manner, while CLASP2 retains its MT-associated localization.

**FIG 4:**
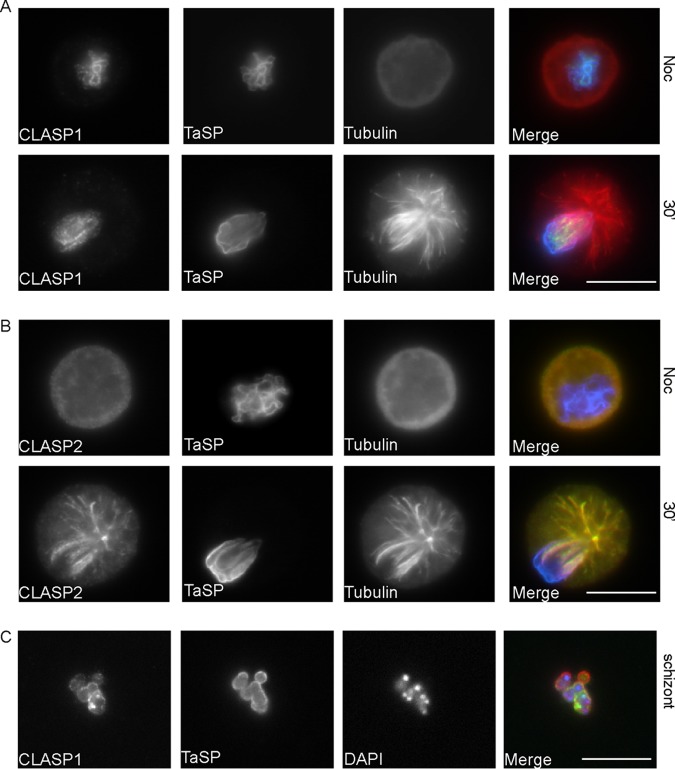
CLASP1 decorates the schizont surface in the absence of microtubules. (A) *T. annulata-*transformed cells (TaC12) were arrested in prometaphase by treatment with nocodazole (Noc) (top panel). The nocodazole was washed away, and MTs were allowed to depolymerize for 30 min prior to fixation. Cells were indirectly labeled with mouse antitubulin (red) and rat anti-CLASP1 (green), and the schizont surface is labeled with rabbit anti-TaSP (blue). Scale bar, 10 μm. (B) TaC12 cells were treated as described above and indirectly labeled with mouse antitubulin (red) and rat anti-CLASP2 (green), and the schizont surface was labeled with rabbit anti-TaSP (blue). Scale bar, 10 μm. (C) *T. annulata* schizonts were purified from their host cell and analyzed with rat anti-CLASP1 (green) and rabbit anti-TaSP (red) antibodies. Schizont nuclei are labeled with DAPI (blue). Scale bar, 10 μm.

To further analyze the mode of CLASP1 association with the schizont, we performed MT depolymerization assays as described previously (13, 14). Even in the absence of MTs, CLASP1 was found to decorate the schizont surface (Fig. 4A). Careful analysis of TaC12 cells as they progressed through mitosis confirmed that CLASP1 interacts with the schizont regardless of the cell cycle stage of the host (Fig. S4A). Even following schizont purification and isolation from the host cells, the parasite surface remained positive for CLASP1 (Fig. 4C).

10.1128/mSphere.00215-17.4 FIG S4  CLASP1 binds to the schizont in the absence of MTs, while CLASP2 associates with the schizont surface in an MT-dependent manner. *T. annulata-*transformed cells (TaC12) were arrested in prometaphase by treatment with nocodazole as shown in Fig. 2. The nocodazole was washed away, and MTs were allowed to repolymerize for up to 120 min prior to fixation. Representative cells in metaphase or anaphase are shown. Cells were labeled with antitubulin (red) and anti-CLASP1 or CLASP2 (green), and the schizont surface is labeled with anti-TaSP (blue). Download FIG S4, TIF file, 1.8 MB. Copyright © 2017 Huber et al. 2017 Huber et al. This content is distributed under the terms of the Creative Commons Attribution 4.0 International license.

The proper localization and function of CLASP1 is crucial for proper progression through mitosis (20), so we were surprised to see that CLASP1 was completely sequestered at the parasite surface. This led us to explore the hypothesis that the closely related CLASP2 might compensate for CLASP1 function in TaC12 cells. Most reports suggest that CLASP1 and CLASP2 have largely identical localizations and redundant roles in mammalian cells. The two proteins have overlapping localizations in HeLa cells, decorating MT plus ends, the Golgi apparatus, and the centrosome in interphase cells (18, 24) and decorating MT plus ends within the spindle, spindle poles, kinetochores, spindle midzone, and midbody during mitosis and cytokinesis (20, 21). In interphase, CLASP1 and CLASP2 play redundant roles in the regulation of MT stability, and it has been proposed that the main function of CLASPs is to promote MT rescue at the cell periphery, increasing MT life span and minimizing MT polymerization (18). We found that in TaC12 cells in the absence of MTs, CLASP2 is dispersed in the cytoplasm and is not detectable on the parasite. Upon polymerization of MTs, CLASP2 localized along the lengths of MTs and associated with the schizont with the same dynamics as we previously reported for tubulin (Fig. 4B and S4B and see reference 13). Thus, CLASP2 associates with the schizont in an MT-dependent manner, and it seems likely that cytoplasmic CLASP2 in *Theileria-*infected cells compensates for the sequestration of CLASP1 at the schizont surface.

### CLASP1 interacts (directly or indirectly) with the schizont protein p104 (TA08425).

The almost complete sequestration of CLASP1 at the schizont surface meant that we could use CLASP1_1256−1538_ to facilitate proximity-dependent biotin identification (BioID) (28) with the aim of identifying the CLASP1 binding partner and other potential host-parasite interactions at the schizont surface. We therefore fused a promiscuous biotin ligase, BirA*, to CLASP1 and expressed it in TaC12 cells using lentiviral transduction. Upon addition of biotin to the culture, proteins in close proximity to CLASP1 were biotinylated and could be affinity purified and identified by mass spectrometry. The complete results of our BioID experiments are beyond the scope of this paper and will be described fully elsewhere (S. Huber and K. Woods, unpublished data). However, two parasite proteins, p104 (TA08425) and TA03615 were identified in this experiment as potential CLASP1-proximal proteins and were therefore chosen for further analysis.

**FIG 5:**
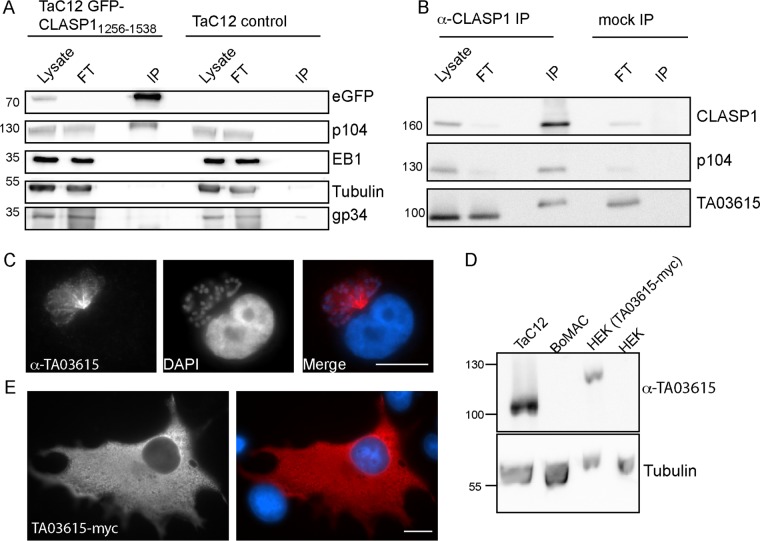
CLASP1 interacts with the schizont surface proteins p104 and TA03615. (A) Nontransduced TaC12 (control) and TaC12 (GFP-CLASP1_1256−1538_) cells were lysed and subjected to coimmunoprecipitation (co-IP) assays using GFP-TRAP magnetic beads. In each case, the soluble lysate (5% of total) flows through following purification (FT), and 20% of the total immunoprecipitations (IP) were analyzed by Western blotting. Analysis with anti-enhanced-GFP (eGFP) antibodies confirmed the expression of the fusion protein and the successful precipitation with GFP-TRAP beads. A signal was obtained with anti-p104 in the GFP-CLASP1_1256−1538_ IP that was absent from the control, indicating that p104 interacts with CLASP1_1256−1538_. Host cell EB1, tubulin, and parasite gp34 were not found in the GFP-CLASP1_1256−1538_ IP. (B) TaC12 cells were subjected to immunoprecipitation with rabbit polyclonal anti-CLASP1 antibodies or rabbit IgG (mock). The lysate (5% of total), flow through (FT), and the immunoprecipitation (IP) (20% of the total amount) were analyzed by Western blotting with anti-CLASP1 (top), anti-p104 antibodies (MAb 1C12) (middle), and anti-TA03615 (bottom), demonstrating the interaction between CLASP1, p104, and TA03615. (C) TaC12 cells were indirectly labeled with affinity-purified rat anti-TA03615 antibodies (red) and analyzed by wide-field microscopy. Host and schizont nuclei are labeled with DAPI (blue). Scale bar, 10 μm. (D) The specificity of the affinity-purified rat anti-TA03615 antibody was confirmed by Western blot analysis. A signal of approximately 100 kDa (TA03615 has a predicted size of 90.5 kDa) was detected in lysates from TaC12 cells but was absent from noninfected bovine macrophages (BoMac) or HEK 293T cells. A signal of approximately 120 kDa could be detected in lysates from HEK 293T cells following transfection with TA03615-myc (predicted size, 92.5 kDa). Antitubulin served as a loading control. (E) TA03615-myc was transiently expressed in BoMac and analyzed by indirect immunofluorescence with mouse anti-myc antibodies (red), revealing no localization to microtubule plus ends. DNA is labeled with DAPI (blue). Scale bar, 10 μm.

Schizont proteins that are expressed on the schizont surface or secreted into the host cell cytoplasm have the potential to be involved in host-parasite interactions. We previously showed that p104 (TA08425) is a major schizont surface protein and that it interacts with host cell EB1 via an SxIP motif embedded in its proline-rich C-terminal domain (14). The identification of p104 in our mass spectrometry data prompted us to perform coimmunoprecipitation (co-IP) experiments to test for an interaction between p104 and CLASP1. First, we analyzed TaC12 cells expressing GFP-CLASP1_1256−1538_. Cells were lysed and subjected to IP using GFP-Trap beads (Fig. 5A). As a control, nontransduced TaC12 cells were subjected to the same treatment. The monoclonal antibody 1C12 was used to detect p104 as described previously (14) and revealed endogenous p104 in the GFP-CLASP1_1256–1538_ IP. The *Theileria* schizont surface protein gp34 (29) and bovine EB1 and tubulin were not identified in the IP, indicating that schizont surface-associated proteins were unlikely to bind nonspecifically to the GFP-CLASP1_1256−1538_-coated beads. Although EB1 is a known interaction partner of CLASP1, this result fits with our observations, considering that the parasite-binding fragment of CLASP1 does not contain the SxIP motifs required for EB1 interaction. Further confirmation for the (direct or indirect) interaction of p104 and CLASP1 was obtained by the co-IP of p104 with endogenous CLASP1 using a rabbit monoclonal anti-CLASP1 antibody (Fig. 5B). Superresolution microscopy showed that fractions of p104 and CLASP1 colocalize on the schizont surface (Movie S4). The interaction between p104 and CLASP1 could be demonstrated only within parasitized cells. Coexpression of p104 with CLASP1 in noninfected mammalian cells, such as COS-7 or HEK 293T cells, did not result in any notable colocalization of the two proteins, nor could the tagged proteins be coprecipitated from transfected cells (data not shown). One possible explanation for this may be that the conformation of membrane-bound p104 is important to facilitate the interaction with CLASP1 and that overexpression of the soluble form of the protein does not allow protein-protein interaction.

10.1128/mSphere.00215-17.9 MOVIE S4  *T. annulata-*transformed cells (TaC12) were analyzed by superresolution microscopy following fixation and staining with anti-p104 (1C12 MAb) (red) and anti-CLASP1 (green). The movie shows a projection through 29 z-stacks captured with a DeltaVision OMX Blaze system. Download MOVIE S4, MOV file, 0.8 MB. Copyright © 2017 Huber et al. 2017 Huber et al. This content is distributed under the terms of the Creative Commons Attribution 4.0 International license.

### TA03615 **is associated with the parasite surface and coprecipitates with CLASP1 and p104.**

Relatively few *Theileria* proteins that have the potential to interact with the host cell have been described so far. The discovery of TA03615 in our BioID data set indicated that this protein might come into contact with host cell proteins and led us to perform an initial analysis on its localization and its possible interaction with CLASP1 and p104. We therefore raised and affinity purified polyclonal antibodies against GST-TA03615_400–550_ and used these antibodies to detect endogenous TA03615 in CLASP1 IPs along with p104. These data indicate that bovine CLASP1 interacts with p104 and TA03615 in *Theileria*-infected cells, although the data do not allow us to differentiate between a direct or indirect interaction. To test the hypothesis that TA03615 may act as a “bridge” protein in the interaction between p104 and CLASP1, we cotransfected tagged TA03615 with p104 and/or CLASP1 in COS-7, HEK 293T, and BoMac cells for colocalization and co-IP experiments, but in no case did we obtain evidence for an interaction between the three proteins (data not shown). These data suggest that the correct endogenous conformation of TA03615 and p104 is required to allow interaction with CLASP1 and that expression of soluble forms of these proteins is not sufficient to maintain an interaction. An alternative explanation may be that, unlike p104-EB1 and p104, CLASP1 and TA03615 do not directly interact with one another, and an intermediate *Theileria*- or bovine-gene-encoded protein is required.

TA03615 is described in GeneDB as a hypothetical protein with a molecular mass of 90.5 kDa. It comprises a predicted N-terminal signal peptide, consists mainly of ordered globular domains, and does not have any predicted transmembrane domains or membrane-anchoring sequences. The protein sequence contains an SxIP-like motif (TFLP), which is surrounded by serines, prolines, and basic residues and thus fulfills the requirements of a putative EB1-binding protein (30). A search on http://blast.ncbi.nlm.nih.gov revealed one FAINT domain (31) and a sequence identity of 54% to a homologous protein in *T. parva* (RefSeq accession no. TP03_0263). IFA with affinity-purified polyclonal rat anti-TA03615 antibodies revealed that this protein localized to the schizont surface, suggesting that it is either secreted and then recruited to the schizont surface or anchored to the membrane (Fig. 5C). A protein of approximately 100 kDa that was absent from BoMac was detected in TaC12 cells following Western blotting, indicating that the antibody specifically recognizes the *Theileria* protein. Detection of TA03615-myc following transfection of HEK 293T cells further supported the specificity of this antibody (Fig. 5D). Considering the presence of an SxIP-like motif in TA03615 and the interaction of EB1 with the parasite, we expressed a soluble form of the protein lacking the signal peptide sequence in COS-7 cells. The fusion protein was distributed throughout the cytoplasm, and no MT plus-end localization or association with other host cell structures was observed, suggesting that it does not interact with EB1 or other +TIPs (Fig. 5E).

## DISCUSSION

In order to facilitate its expansion throughout the body of an infected animal, *Theileria* has evolved differently from other parasites. While *Toxoplasma* and *Plasmodium* ensure their spread throughout their host animals via the production of thousands of invasive parasite forms (tachyzoites or merozoites) which burst from the host to subsequently invade new cells, the *Theileria* schizont utilizes a mechanism that allows it to disseminate throughout the body of the infected animal without ever having to leave the confines of its harboring cell. Transformed *Theileria*-infected cells proliferate continually and become invasive and metastatic in a process driven by matrix metalloproteinase (*mmp9*) expression (9, 12). The presence of a viable schizont in the cytoplasm of an infected cell is critical to maintain this transformed phenotype. By recruiting and stabilizing host cell MTs at the surface of the cell, the *Theileria* schizont integrates itself into the host cell’s own mitotic machinery (13). This allows the schizont to passively “hitch a ride” as the immortalized host cell divides and results in the clonal expansion of infected cells.

Over the past few years, we have attempted to understand the molecular mechanisms underlying the interaction between *Theileria* and host cell MTs. Recently, we described the interaction between the parasite and host cell +TIP EB1, mediated via a classic EB1-binding SxIP motif harbored by the schizont membrane protein p104 (14). The +TIP network is a highly complex web of dynamically interacting proteins that consists of a large number of structurally and functionally unrelated proteins, most of which are recruited to MT plus ends via an interaction with EB1 (32). A likely explanation for the complexity of the +TIP network is that it facilitates the spatiotemporal regulation of +TIP activity at MT plus ends, and it seems that *Theileria* has tapped into this dynamic network by hijacking the central regulator of the network. We previously used kymograph analyses to demonstrate that parasite-associated astral MTs are highly stable in comparison to cytoplasmic MTs (13). EB1 does not possess MT-stabilizing properties, however, and so its association with the parasite surface is unlikely to explain this observation. We hypothesized that one function of parasite-associated EB1 might be to recruit MT-stabilizing proteins, such as CLASP1, to the schizont surface. However, here we show that CLASP1 binds to the schizont in an EB1-independent manner via its kinetochore-binding domain. The association of CLASP1, which harbors two SxIP motifs, with the *Theileria* surface provides a possible explanation for why we were previously unable to abolish the interaction between EB1 and the parasite using competitive SxIP-containing peptides and hints at the evolutionary importance of EB1 association with *Theileria*. In this work, we also describe a previously uncharacterized *Theileria* protein, TA03615, which is expressed on the schizont surface and also contains an SxIP motif, although under the conditions tested here we could not detect any plus-end tracking behavior.

CLASPs are thought to promote the polymerization of specific subsets of MTs and link them to cellular structures (33). Studies of *Schizosaccharomyces pombe* have shown that CLASPs can also promote MT rescue and suppress catastrophes independently of other proteins (34). CLASP1 is recruited to kinetochores during mitosis, where it regulates kinetochore-MT dynamics and plays an essential role in mitotic progression (20, 22). It seems likely that parasite-associated CLASP1 plays a similar role in stabilizing MTs at the schizont surface. CLASP1 and CLASP2 have been widely reported to have overlapping and redundant functions during interphase and mitosis (18, 21), and so the distinct localization of CLASP1 and -2 in *Theileria*-infected cells was striking. Depletion experiments with human cells revealed that CLASP1, but not CLASP2, is required for correct positioning of the spindle in dividing human cells, and it was proposed that CLASP1 functions in the initial capture and stabilization of astral MTs at the cell cortex (19, 35). We hypothesized that CLASP1 functions at the schizont surface to stabilize MTs and perhaps to recruit components of the central spindle, such as PRC1 (36), thus ensuring the incorporation of the schizont within the central spindle during host cell division. Unfortunately, we could not obtain any data to support this hypothesis because partial depletion of CLASP1 had no impact on parasite association with MTs or segregation during host cell division, and complete knockdown of CLASP1 in parasitized cells failed. Disruption of CLASP1 function with a dominant negative mutant (20) in *Theileria-*infected cells caused MT bundling and induced a dramatic alteration in parasite size, morphology, and position, emphasizing how important proper MT dynamics are for *Theileria* infection. CLASP1 has also been implicated in other important host-pathogen interactions. Infection with herpes simplex virus 1 (HSV-1) induces the stabilization of MTs in human fibroblasts in a CLASP1-dependent manner. While depletion of CLASP1 or CLASP2 had no effect on virus entry into cells, the production of infectious viral particles was reduced and cell-cell spread of the virus was impaired (37). CLASPs were also implicated in *Trypanosoma cruzi* infection, with GFP-CLASP1/2 accumulating somewhat at the site of trypomastigote entry. Silencing of CLASP1, but not CLASP2, in HeLa cells impaired *T. cruzi* entry while having no impact on *Toxoplasma gondii* infection (38).

We have previously described how host-parasite interactions in *Theileria*-infected cells, as well as the phosphorylation of schizont surface proteins, vary in a host cell cycle-dependent manner (39). Plk1 associates with the schizont specifically during G_2_ phase and anaphase, and we showed that its kinase activity is important for the association of central spindle MTs with the parasite (13). EB1 is conspicuously absent from the schizont during metaphase, and as with Plk1, its interaction with the schizont is negatively regulated by Cdk1 activity (14). In contrast to Plk1 and EB1, CLASP1 associates with the parasite surface throughout the cell cycle, even remaining bound following isolation of the schizonts from the host cell, and is almost undetectable in the host cell cytoplasm. Considering that genetic manipulation of the *Theileria* schizont is not yet possible, the analysis of protein-binding partners is an important tool for discovering the function of parasite-encoded proteins. Our data reveal that p104 not only is a binding partner of host cell EB1 as we reported before but also interacts, possibly indirectly, with CLASP1. This indicates that p104 is of major importance in the binding of MTs to the parasite and, thus, for the maintenance of the transformed phenotype and for achieving clonal expansion. Further, we have described for the first time TA03615, a previously noncharacterized schizont protein that localizes to the parasite surface and coimmunoprecipitates with p104 and CLASP1, indicating that it might play a role in mediating *Theileria*-MT interactions.

## MATERIALS AND METHODS

### Cell culture, drug treatment, and transfections.

TaC12 (*T. annulata*-infected bovine macrophages), BoMac (a simian virus 40 [SV40]-transformed bovine macrophage line), and HEK 293T cells were cultured as described previously (13, 14). *T. annulata-*infected peripheral blood mononuclear cells (clone TaD7) and *T. parva*-infected T cells (Tp_951T_F31) were kind gifts from Brian Shiels and Declan McKeever and were cultured as previously described (40, 41). Schizonts were isolated from TaC12 cells using a protocol modified from reference 42 that has been described in detail elsewhere (39). Briefly, host cells were perforated using activated aerolysin and subjected to mechanical lysis, and schizonts were separated from host cell debris and nuclei by using a Nycodenz step gradient. For the MT repolymerization assay, cells were incubated in 0.1 µg/ml nocodazole (BioTrend) for 16 h and harvested by shake-off as previously described (13). HEK 293T cells were transfected with Lipofectamine 2000 (Invitrogen) by following the manufacturer’s instructions, and BoMac and TaC12 cells were electroporated using Amaxa four-dimensional (4-D) nucleofection (Lonza; cell solution SF, program DS103).

### Lentiviral transduction and FACS sorting.

Lentiviruses were produced by transfection of HEK 293T cells with a third-generation lentiviral transfer vector (pRRL-RSrII) containing the gene of interest, a second-generation packaging vector (psPAX2), and a vesicular stomatitis virus G (VSV-G) coat envelope vector (pMD2.G) in a 5:3:2 ratio using FuGENE HD transfection reagent (Roche). The medium was exchanged 24 h after transfection, and lentiviral-particle-containing medium was collected 48 h and 72 h posttransfection and filtered. Transduction was performed by incubating 2 × 10^5^ TaC12 cells with 2 ml of the virus-containing medium. Cells were transduced three times for 24 h, with a recovery time of 24 h between the transduction steps. To enrich TaC12 cells expressing GFP-CLASP1_1256−1538_, cells were sorted using Aria III (BD Biosciences) fluorescence-activated cell sorting (FACS) in the Department of Clinical Research at the University of Bern.

### Sporozoite infection.

A Holstein calf was infected by subcutaneous inoculation with 1 × 10^6^ cells infected with *T. annulata* A21/AT1. The calf was monitored for the progress of infection by taking rectal temperature and examining lymph node biopsy specimens and blood smears for the presence of schizonts and piroplasms, respectively. Unfed nymphal *Hyalomma excavatum* ticks were applied to the ears of the calf when first piroplasms were detected in the blood smears. After the ticks detached and molted, the sporozoite stabilate A21/AT1 was prepared as described in reference 43.

Peripheral blood mononuclear cells were isolated from venous bovine blood taken from a jugular vein by flotation on Ficoll-Paque as described previously (44). Cells were resuspended in RPMI 1640 medium (containing 2 mM glutamine, 5 × 10^−5^ M 2-mercaptoethanol, 100 IU/ml penicillin, 100 μg/ml streptomycin, and 10% fetal calf serum [FCS]) to obtain 10^7^ cells/ml. Five hundred microliters of cell suspension was mixed with an equal volume of *T. annulata* stabilate A21/AT1 diluted in culture medium to obtain an equivalent of one tick per milliliter. The suspension was incubated for 30 min at 37°C with occasional agitation and then centrifuged at 200 × *g* for 5 min and washed twice in phosphate-buffered saline (PBS). The cells were fixed with 4% paraformaldehyde (PFA) in PBS for 10 min at room temperature, washed twice, and stored in PBS containing 0.02% sodium azide. Infections were performed at Adnan Menderes University, Turkey. See ethical statement 64583101/2013/018 from the Animal Ethics Committee of Adnan Menderes University for regulations used regarding the care and use of laboratory animals.

### Expression constructs.

The following plasmids were purchased from Addgene: the pLKO1 shRNA cloning vector (10878), pRRL-RSrII (12252), second-generation packaging vector (psPAX2, 12260), and VSV-G coat envelope vector (pMD2.G, 12259; Tronolab). The following plasmids were kind gifts: pEGFPC1-CLASP1 from Anna Akhmanova (24) and GFP-CLASP1_250–943_, CLASP1_1–270_, and GFP-CLASP1_1256−1538_ from Helder Maiato (20). The minimal parasite-binding domain of CLASP1 was amplified by PCR from the GFP-CLASP1_1256−1538_ plasmid and cloned into pEGFP-C1 using BamHI and HindIII sites. For generation of lentiviral particles, GFP-CLASP1_1256−1538_ and GFP-CLASP1_250–942_ were subcloned into RsrII restriction sites of the pRRL_RsrII transfer vector. The oligonucleotides coding for CLASP1 shRNA were annealed and inserted into the AgeI and EcoRI restriction sites of pLKO1. For generation of GST fusion protein for antibody production, TA03615_400–550_ was amplified by PCR from TaC12 genomic DNA (gDNA) and cloned into EcoRI and XhoI restriction sites of the pGEX-6P-2 vector. For expression in mammalian cells, full-length TA03615 lacking the signal peptide was cloned into the pEF-myc/His vector using NotI and XbaI restriction sites. Primers are provided in Table S1 in the supplemental material.

10.1128/mSphere.00215-17.5 TABLE S1  List of primers. Download TABLE S1, DOC file, 0.1 MB. Copyright © 2017 Huber et al. 2017 Huber et al. This content is distributed under the terms of the Creative Commons Attribution 4.0 International license.

### Antibodies.

The following antibodies were used: rat monoclonal antibody (MAb) anti-CLASP1 (KT66; Absea Biotechnology), anti-CLASP2 (KT69; Absea), and anti-GFP (3H9; Chromotek), rabbit MAb anti-CLASP1 (2996-1; Epitomics), mouse MAb c-Myc (9E10; Santa Cruz) and anti-alpha tubulin (clone DM1A, T9026; Sigma), rat MAb anti-EB1 (clone KT51 [Absea Biotechnology], IgG from rabbit serum [I5006; Sigma], anti-p104 [MAb 1C12, a kind gift from Brian Shiels {45}]), rabbit polyclonal antibody (PAb) anti-TaSP (generated by the laboratory of Isabel Roditi, Bern, Switzerland, using the procedure described by Schnittger et al. [46]), and rat PAb anti-gp34 (29). GST-TA03615_400–550_ was expressed in BL21 Star *Escherichia coli* (Invitrogen), purified using glutathione-Sepharose beads (GE Healthcare), and used for the generation of anti-TA03615 antibodies. Anti-TA03615 antibodies were precleared against *E. coli* and BoMac lysates before being affinity purified with the purified antigen.

### Cell lysis and coimmunoprecipitation.

TaC12 cells and transduced TaC12 cells expressing the GFP_CLASP1_1256−1538_ construct were lysed in lysis buffer containing 0.5% NP-40 and subjected to GFP-TRAP coimmunoprecipitation (co-IP) (Chromotek) by following the manufacturer’s instructions. For IP of endogenous CLASP1 from TaC12 cells, 1 μg rabbit MAb, anti-CLASP1 antibody, or IgG from rabbit serum (I5006; Sigma) (negative control) was bound to 20 μl protein G magnetic Dynabeads (Life Technologies, Inc.) and incubated with 1 mg TaC12 lysate (lysis buffer consisted of 50 mM Tris-HCl, pH 7.4, 150 mM NaCl, 1 mM EDTA, 1% NP-40, 0.25% sodium deoxycholate, phosphatase, and protease inhibitors) overnight at 4°C. The beads were washed 3 times with lysis buffer and 3 times with PBS prior to elution of protein complexes by boiling them in Laemmli SDS sample buffer.

### Immunofluorescence analysis and time-lapse imaging.

Cells were grown on coverslips and fixed with 4% paraformaldehyde for 10 min at room temperature, followed by permeabilization in 0.2% Triton X-100 as described previously (13, 14). Antibodies were diluted in 10% heat-inactivated FCS in PBS, DNA was stained with DAPI (4′,6-diamidino-2-phenylindole; Invitrogen), and the coverslips were mounted with mounting medium (DAKO). Samples were analyzed on a Nikon Eclipse 80i wide-field microscope with a Hamamatsu Orca R2 camera using 60× and 100× Plan Apo objectives (Nikon) and the Openlab 5 software (Improvision). Fiji was used for image quantification (47), and Photoshop (Adobe) was used to prepare the images. Time-lapse imaging was performed on a TE2000E-PFS microscope (Nikon) using Plan Fluor 60× objectives (Nikon), an Orca-ER charge-coupled device camera (Hamamatsu), and an incubation chamber (Life Imaging Services).
