# Erratum for Huber et al., “The Microtubule-Stabilizing Protein CLASP1 Associates with the *Theileria annulata* Schizont Surface via Its Kinetochore-Binding Domain”

**DOI:** 10.1128/mSphere.00413-17

**Published:** 2017-09-27

**Authors:** Sandra Huber, Romina Theiler, Daniel de Quervain, Olga Wiens, Tulin Karagenc, Volker Heussler, Dirk Dobbelaere, Kerry Woods

**Affiliations:** a Institute for Animal Pathology, Vetsuisse Faculty, University of Bern, Bern, Switzerland; b Institute for Molecular Pathobiology, Vetsuisse Faculty, University of Bern, Bern, Switzerland; c Department of Parasitology, Adnan Menderes University, Faculty of Veterinary Medicine, Isikli, Aydin, Turkey; d Institute of Cell Biology, University of Bern, Bern, Switzerland

## ERRATUM

Volume 2, no. 4, e00215-17, 2017, https://doi.org/10.1128/mSphere.00215-17. Tulin Karagenc’s name was misspelled in the author byline. The correct byline appears above.

