# Supplementary figures and images for: The Microtubule-Stabilizing Protein CLASP1 Associates with the Theileria annulata Schizont Surface via Its Kinetochore-Binding Domain
Source: mSphere. 2017 Aug 23;2(4):e00215-17. doi: 10.1128/mSphere.00215-17 (PMC5566832; doi:10.1128/mSphere.00215-17)

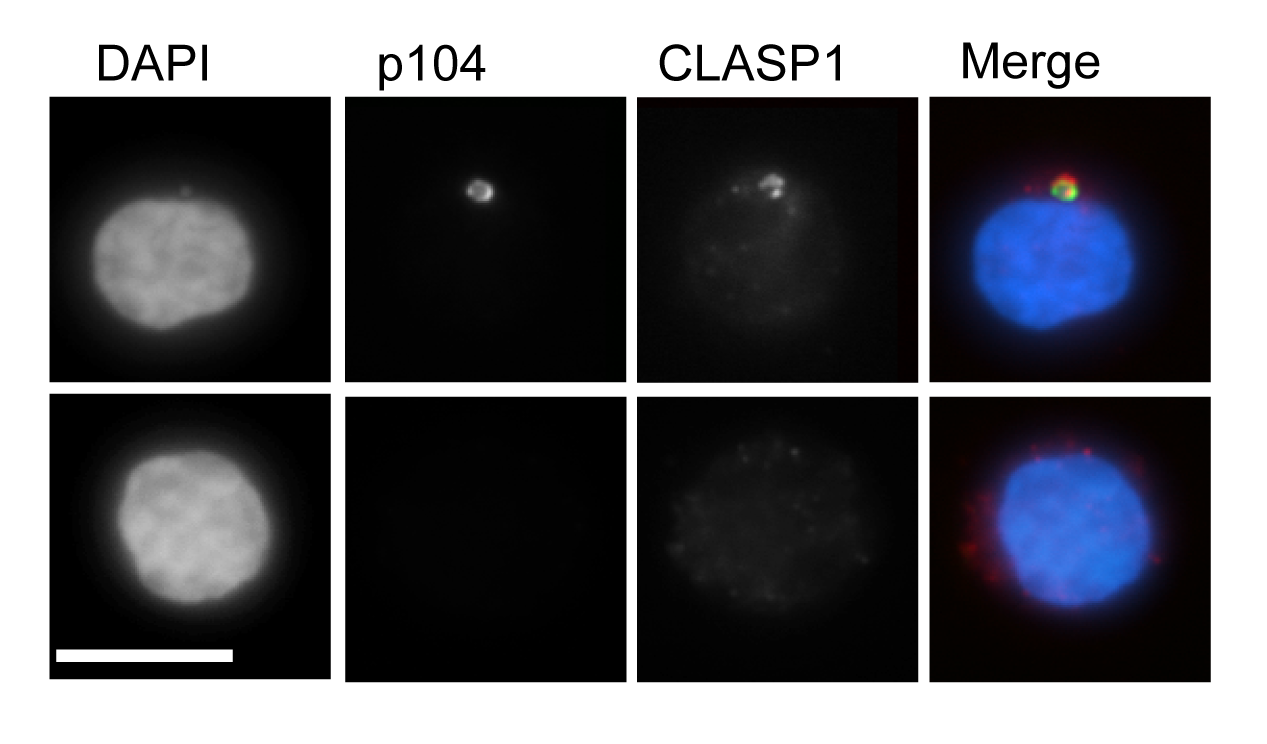

Supplement: FIG S1 [file sph004172349sf1.tif]

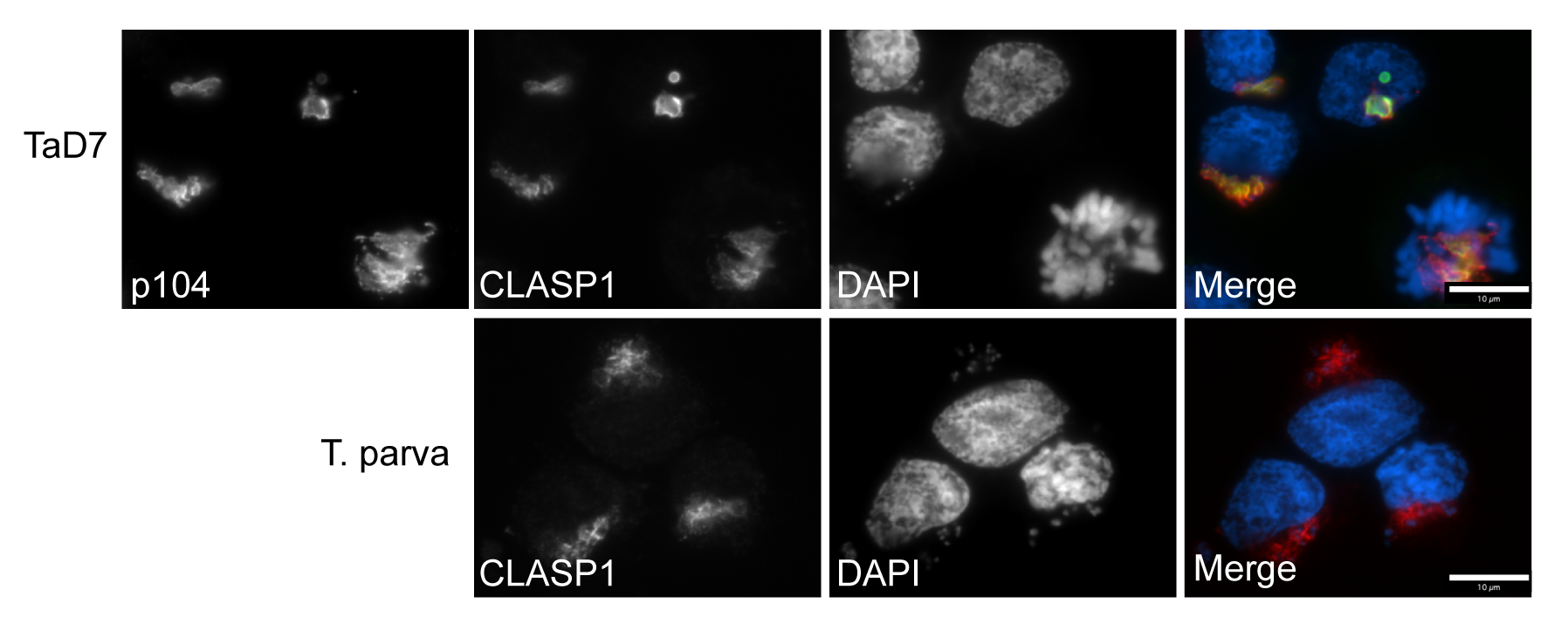

Supplement: FIG S2 [file sph004172349sf2.tif]

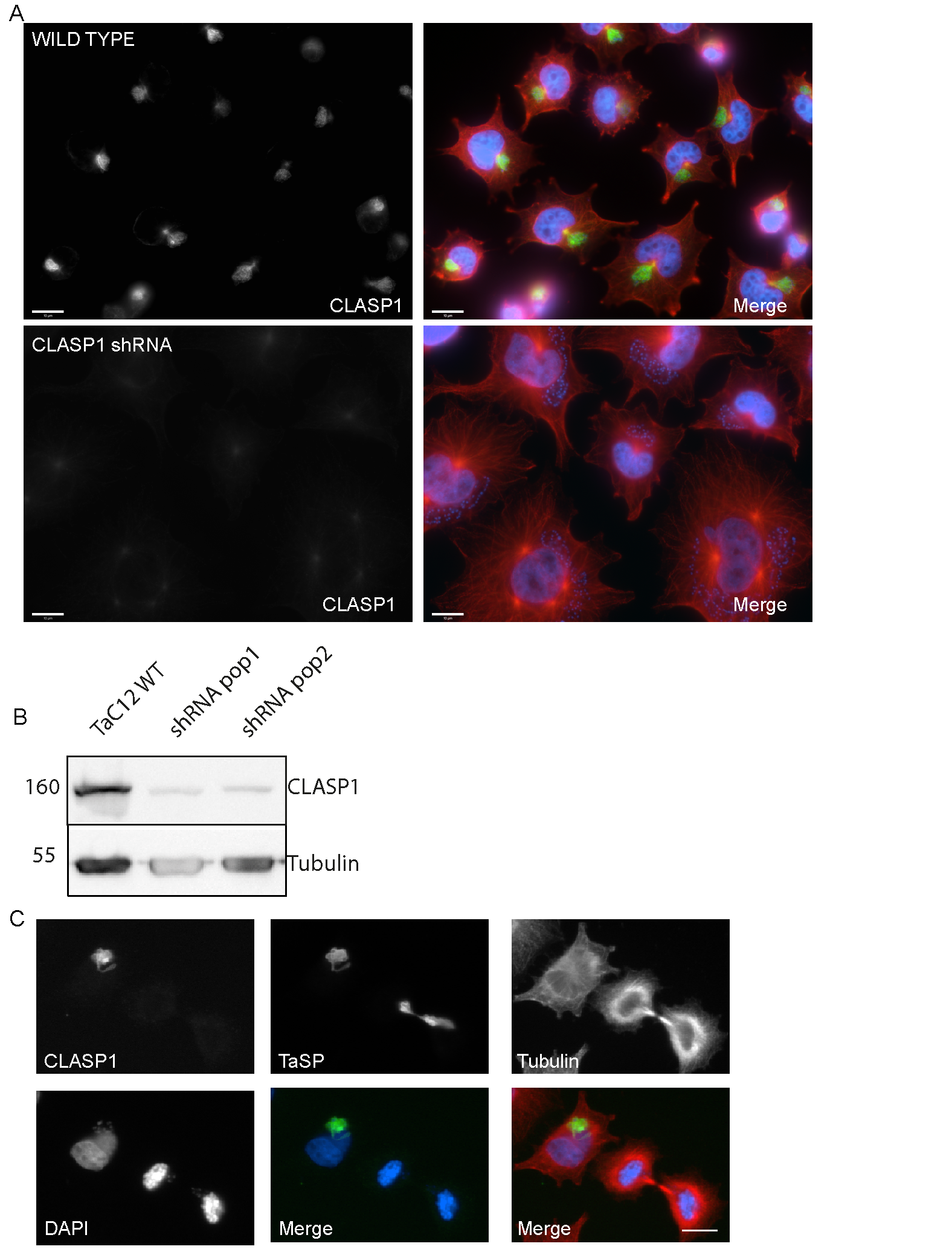

Supplement: FIG S3 [file sph004172349sf3.tif]

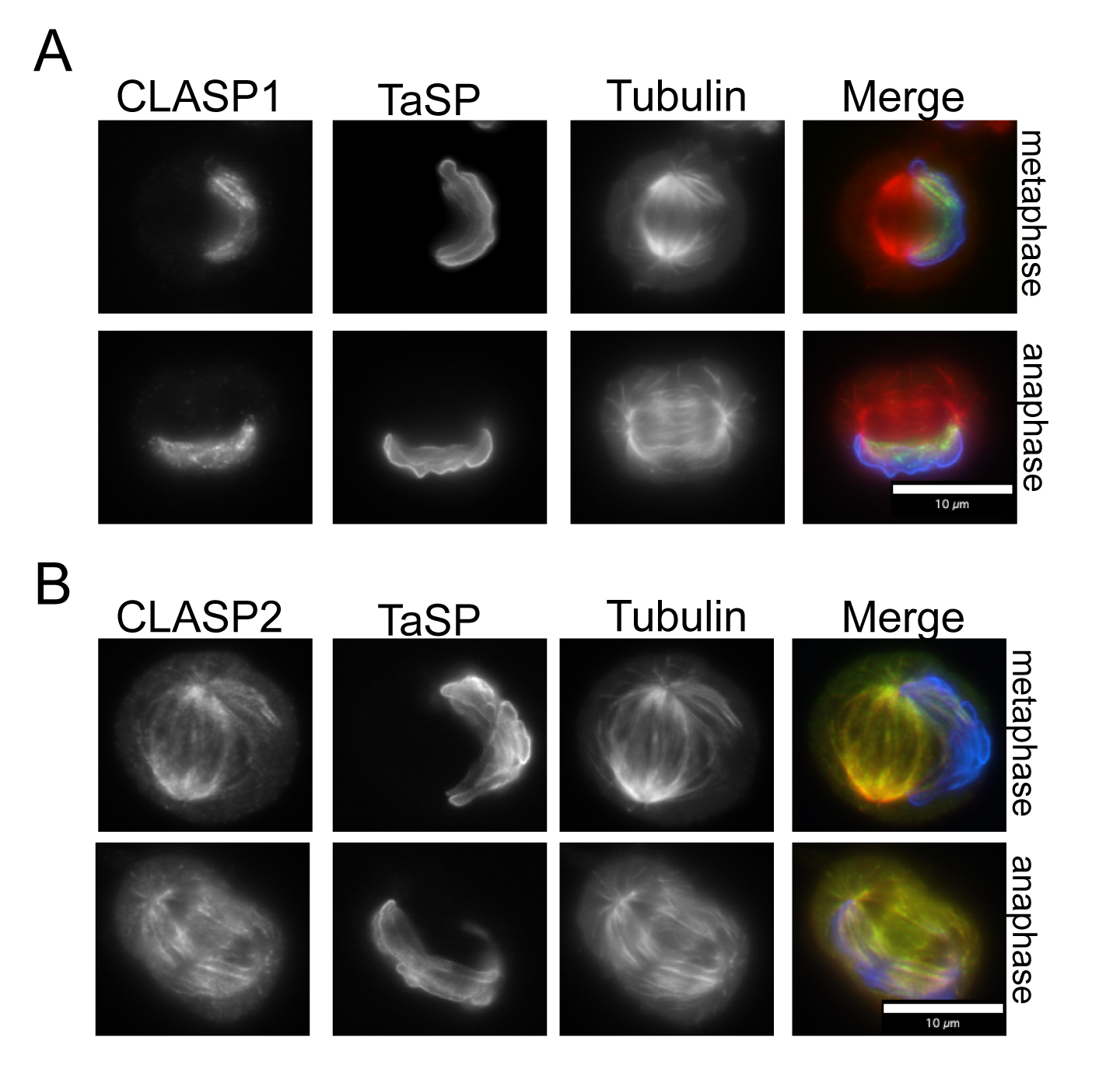

Supplement: FIG S4 [file sph004172349sf4.tif]
