# Supplementary material for: The Microtubule-Stabilizing Protein CLASP1 Associates with the Theileria annulata Schizont Surface via Its Kinetochore-Binding Domain
Source: mSphere. 2017 Aug 23;2(4):e00215-17. doi: 10.1128/mSphere.00215-17 (PMC5566832; doi:10.1128/mSphere.00215-17)
Supplement: TABLE S1 [file sph004172349st9.docx]

Supplementary Table 1_ List of Primers

| Primer Name | Sequence | Restriction Enzyme |
| --- | --- | --- |
| CLASP1 MT lenti rev | ATATCGGTCCGTTATAATTGTGTGAGAAGAAC | *Rsr*II |
| CLASP1 lenti rev | ATATCGGTCCGTTAGCTGTGCGTGGAGACATCGG | *Rsr*II |
| EGFP_LV_RsrF | ATATCGGACCGCCACCATGGTGAGCAAGGGC | *Rsr*II |
| CLASP1for3766 | GCAAGCTTCGTTCCCGGGGCCGCGGGCG | *Hin*dIII |
| CLASP1for4093 | GCAAGCTTCGCTGGCGTTAAGAGTTTTG | *Hin*dIII |
| CLASP1rev4391 | GCGGATCCTTAGTCGACAAGGAGCTGCAG | *Bam*HI |
| CLASP1rev4524 | GCGGATCCTTACCCTGTGAGCTGTGCAAG | *Bam*HI |
| CLASP1rev4617 | GCGGATCCTTAGCTGTGCGTGGAGACATC | *Bam*HI |
| 03615_150aa_EcI_F | ATATGAATTCAATACCTAGAATGTCTCGAACG | *Eco*RI |
| 03615_150aa_Xho_R | ATATCTC GAGTTATGCCATAAAAACTCTTTT TAAAAC | *Xho*I |
| TA03615_Not1_For | ATATGCGGCCGCACCATGGACCCTAAAGTTTTAAAC | *Not*I |
| TA03615_Xba1_Rev | ATATTCTAGACTATCATCCTCGTCGATGTC | *Xba*I |
| SHRNA1_CLASP1_F | CCGGATGACCAGACCATGTTAGATAAACTCGAGTTTATCTAACATGGTCTGGTCATTTTTTG | *Age*I*, Eco*RI |
| SHRNA1_CLASP1_R | AATTCAAAAAATGACCAGACCATGTTAGATAAACTCGAGTTTATCTAACATGGTCTGGTCAT | *Age*I*, Eco*RI |
